# The “Secret of Seven Stones”: Short-Term Efficacy of an Online Intergenerational Sexual Health Education Game for Early Adolescents and Their Parents

**DOI:** 10.1177/2161783X251370416

**Published:** 2025-09-10

**Authors:** Ross Shegog, Christine Markham, Melissa Peskin, Robert C. Addy, Sara Dube, Diane Santa Maria, Susan Tortolero Emery, Johnny M. Wilkerson, Elizabeth Baumler, Laura Armistead, Pooja Chaudhary, Hsing-Yi Song, Angela Spencer, Jeffery McLaughlin

**Affiliations:** 1 Department of Health Promotion and Behavioral Sciences, UTHealth Houston School of Public Health, Houston, Texas, USA.; 2 The Widen Lab, University of Texas at Austin, Austin, Texas, USA.; 3 University of Texas Health Science Center at Houston School of Nursing, Houston, Texas, USA.; 4 University of Texas Health Science Center at Houston School of Behavioral Health Sciences, Houston, Texas, USA.; 5 Mathematica Policy Research, Princeton, New Jersey, USA.; 6 University of Texas Health Science Center at Houston School of Biomedical Informatics, Houston, Texas, USA.; 7 Special Supplemental Nutrition Program for Women, Infants, and Children (WIC), Washington, District of Columbia, USA.; 8 Radiant Digital, Vienna, Virginia, USA.

**Keywords:** serious games, sexual health, parent–child communication, intergenerational games, sexually transmitted infections, teenage pregnancy

## Abstract

**Objectives:**

Intergenerational games offer a potential channel to impact parent–youth sexual health communication. The “Secret of Seven Stones” (SSS) is an 18-level online adventure game and parent website designed to engage parents and youth (11–14 years) in conversations about healthy dating relationships and sexual behavior and to provide sexual health skills training to youth. Study hypotheses were that SSS exposure would increase sexual health parent–child communication, increase youth intentions to delay sexual debut, and reduce youth exposure to situations that promote sexual activity.

**Materials and Methods:**

SSS was evaluated in the homes of parent–youth dyads randomly assigned to intervention (*n* = 40) and comparison (*n* = 45) conditions. Online surveys were used to collect baseline and three-month follow-up data on dyadic sexual health communication, determinants for communication and youth sexual behavior, and game usability ratings.

**Results:**

Dyads comprised parents (*n* = 83, 47% white, 93% female, 44.4 ± 5.8 years) and youth (*n* = 83, 42% white, 54% male, 12.9 ± 1.1 years, and 96% sexually inexperienced). Frequency of parent–youth sexual health communication and youth communication self-efficacy increased in those playing SSS compared with those in the comparison group (*P* < 0.01). Youth perceived parent–youth communication as more open and demonstrated significant improvement in condom and human immunodeficiency virus/sexually transmitted infection knowledge and perceptions of parents’ beliefs about sex (<0.001). Usability ratings were higher on ease, credibility, and helpfulness (all >78%) but lower on duration and appeal (<56%).

**Conclusion:**

This study demonstrated the utility of an in-home intergenerational sexual health education game to impact parent–youth communication by short-term follow-up. Further investigation of longer-term behavioral impact is indicated.

## Introduction

**FIG. 1.**
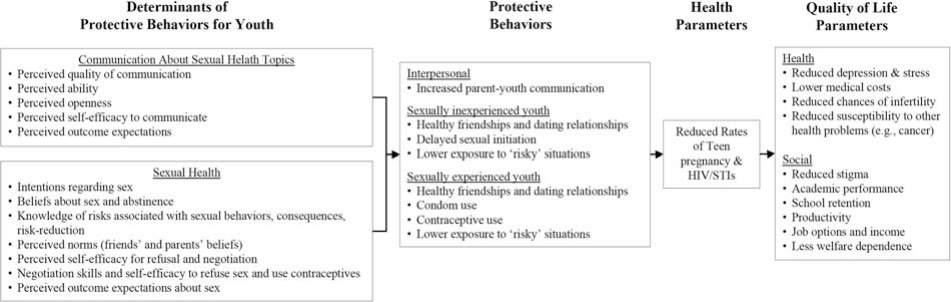
Determinants for parent–youth sexual health communication, delayed sexual initiation, and risk reduction practices*. *Psychosocial determinants, derived from Social Cognitive Theory,^4^ social influence models,^5^ and theory of triadic influence^6^ associated with teen sexual health behaviors.^7,8^

In the United States, adolescent sexual health remains a public health priority. Teen birth rates for youth 15–19 years of age approximate 13.6 births per 1000 females,^1^ adolescents 15–24 years of age account for nearly half of new cases of sexually transmitted infections (STI) annually, and early sexual debut is associated with risky sexual behaviors that can compromise health outcomes.^2,3^ Sexual health programs can promote delayed sexual initiation for sexually inexperienced youth and risk reduction behaviors (e.g., condom use, contraceptive use, human immunodeficiency virus [HIV] and STI testing) for sexually experienced youth. Such programs target psychosocial antecedents to these behaviors and are benefited by contextualizing sexual health within a supportive social network (Fig. 1).^4–8^

Within this social network, parents are critical in influencing youth risk behaviors.^9,10^ Empirically, health interventions involving caregivers demonstrate larger outcome effect sizes compared with those without caregivers.^11^ Parental discussions can reduce risky sexual behaviors,^12,13^ delay sexual initiation, reduce teen pregnancy and STIs, and increase condom and contraceptive use.^14^ Parents are not only open to discussing sexual health topics with their child^15^ but also report barriers of limited knowledge, skills, or time, and feelings of embarrassment.^16,17^ Youth not only desire these discussions but also feel their parents need training on how to communicate about these topics.^18^

School-based sexual health education programs can often improve youth sexual behaviors but provide only superficial parent involvement,^19–23^ while parent-focused and home-based in-person interventions focus on improved parent–child sexual health communication but often require intense parental training that limits reach and implementation.^19,24^ Youth-focused digital interventions can delay sexual initiation and reduce sexual risk behaviors, and such interventions are seen as appealing and relevant by parents and youth,^25,26^ yet parental involvement in these programs remains a challenge.^13,19–22,27–31^

Intergenerational serious gaming may have utility for improved parent involvement in sexual health education.^25,32–34^ An intergenerational game (IGG) for health provides a platform for adults and youth to learn and communicate with each other through collaborative engagement toward achieving a health-related objective. Intergenerational play can improve communication, learning, and emotional bonding between adults and youth, yet there is a dearth of research on the application of IGGs to enhance parent–youth communication and skills training for sexual health.^35,36^

The purpose of this pilot study was to explore the impact of an online sexual health IGG prototype, the “Secret of Seven Stones” (SSS), designed for in-home use by middle-school youth (11–14 years) and their parents. Respective hypotheses for youth and parents to assess primary and secondary outcomes were:

### Youth

Compared with youth in the comparison condition, youth playing SSS will report significantly (1) increased communication frequency about sexual health with their parent; (2) increased intentions to delay sexual initiation (until high school graduation or marriage) and to use condoms if sexually active; (3) reduced exposure to risky situations; (4) improved attitudes, knowledge, and outcomes expectations regarding parental sexual health communication; (5) improved attitudes, knowledge, and perceived norms regarding delayed initiation of sexual behavior; and (6) improved quality of general parental communication.

### Parent

Compared with parents in the comparison condition, parents accessing SSS will report significantly (1) increased communication frequency about sexual health with their youth; (2) improved perceptions of their youth’s intentions toward delayed sexual debut and condom use; (3) improved attitudes, knowledge, and outcomes expectations regarding youth sexual health communication; (4) improved attitudes and knowledge regarding their youth’s delayed initiation of sexual behavior; and (5) improved quality of general communication with their youth.

## Materials and Methods

### Study design and participants

The SSS was evaluated using a randomized, two-group pre- and post-test design in which parent–youth dyads (*n* = 85) were randomly assigned to intervention (*n* = 40) and comparison (*n* = 45) conditions. Inclusion criteria required participants to be English-speaking, that the youth be 11–14 years old, and that parents have mobile phones with short message service, and a home computer with consistent internet access. The study was conducted from Fall 2014 through Fall 2015 in a large, metropolitan area in southeast Texas that was geographically convenient to enable in-person data collection.

### Recruitment and randomization

Interested parent–youth dyads (*n* = 109) contacted the research coordinator in response to community magazine and newspaper advertisements (32 dyads), school presentations (20 dyads), professional meetings (2 dyads), flyers posted in clinics and universities (13 dyads), or parental referral (42 dyads). Of these 109 dyads, 85 agreed to a home visit to obtain parental consent, youth assent, and baseline assessment, followed by simple random assignment of dyads into study conditions based on an a priori random number array. The remaining 24 dyads were ineligible, too busy, unable to commit for the duration, or unresponsive. Retention at 3-month follow-up was 97.6% (83 dyads-39 Intervention and 44 Comparison). Two dyads (one in each study condition) were lost to follow-up due to illness and disinterest, respectively. Incentives for each survey completed were $20 (youth) and $40 (parents). The study was approved by the UTHealth Houston institutional review board (HSC-SPH-12-0584) and the school district’s Office of Research and Accountability.

### Intervention condition

The SSS prototype comprised (1) an adventure game, (2) text messages, and (3) a parent website. The description of SSS game mechanics and components has been published in detail elsewhere.^35^

**FIG. 2.**
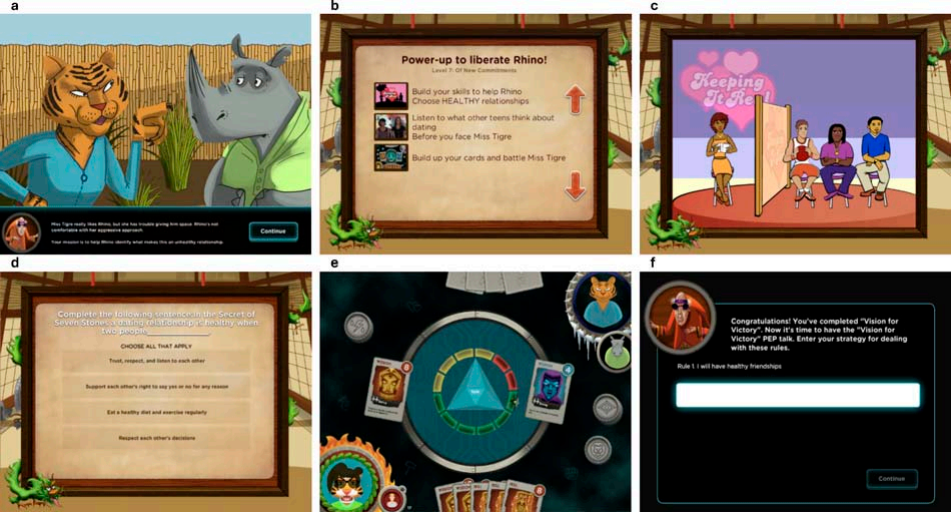
Example “Secret of Seven Stones” game components. **(a)** A “Battle” scenario, the player must help resolve to liberate the characters. **(b)** The “Dojo” selection of skills training exercises that prepare the player for “Battle.” **(c)** An exemplar activity the player must complete before “Battle.” **(d)** A “Challenge” quiz to assure player mastery of content before facing opponents in “Battle.” **(e)** A “Battle” where the player must select the appropriate wisdom, skill, and support cards against the opponent. **(f)** An exemplar “PEP” lock that asks the player their strategies to protect a given personal rule (e.g., “I will only have healthy friendships”). PEP, Partner-Engage-Plan.

In the SSS adventure game, youth progress sequentially through 18 levels (each of 45–60 minutes duration) and win by rescuing 45 citizens in the town of Seven Stones from an evil “boss.”^35^ In a single sequence of play the youth (1) visits a town location; (2) meets citizens who have an interpersonal challenge (e.g., protecting personal rules in the face of peer pressure); (3) visits the “Dojo” and completes educational activities (e.g., animations, mini games, role model videos, and simulations) to build relevant knowledge and skills; (4) completes a “challenge” quiz to earn “wisdom,” “skill,” and “support” battle cards (with higher quiz scores equating to higher value cards); and (5) engages in a card “battle” against the “boss” (Fig. 2). Winning the battle frees the citizen and enables continued game progress. A loss leads to remediation in the dojo to earn stronger cards and a repeat battle.

SSS is founded on social cognitive theory (SCT) and a life-skills self-regulation paradigm (Select, Detect, Protect) where youth *select* personal rules, *detect* challenges, and *protect* their rules.^4–6,20–22^ SCT methods include goal setting (e.g., youth sets personal rules and strategies), enactive mastery and verbal persuasion (e.g., interactive simulations), reinforcement (e.g., mastery of content enables higher value battle cards that enable battle wins and game progress), and modeling (e.g., parent/youth video testimonials describing communication success strategies).^20–22,37,38^ Educational topics comprise communication and negotiation, future goals and personal rules, healthy and unhealthy friendships and dating relationships, puberty and reproduction, consequences of sex, responsible internet use, dating violence prevention, and condom and contraceptive use. Embedded skills training activities (*n* = 179), each approximating 1–12 minutes, include mini-games, peer-modeling videos, fact sheets, virtual role-playing activities, and quizzes.^20–22^

Seven *automatic text messages* were sent to the parents that provides updates on their youth’s progress, cues to initiate a PEP talk (Partner-Engage-Plan) to discuss their youth’s behavioral goals and topics relevant to the educational topics covered by their youth (listed above), and linkage to the relevant activity on the parent website (discussed below). In each PEP talk, dyads *partner* together in a distraction-free environment, *engage* with each other to discuss SSS content, and *plan* strategies to maintain the conversation and protect the youth’s personal rules. After each PEP talk, parents retrieved a code that unlocked the next game level for their youth.

The SSS parent website was designed to increase parents’ involvement in the SSS game and build communication skills and self-efficacy to engage in each PEP talk. Parents could monitor their youth’s progress, adjust SSS content settings, preview game activities, access 15 3-minute peer role model videos describing positive real-world communication experiences, and refer to tip sheets describing strategies to engage youth.

SSS implementation occurred in the home on desktop computers. Trained study staff set up parent website accounts, installed SSS, gave an introductory orientation, and recommended a schedule for frequency and duration of play to enable SSS completion within a three-month testing period.

### Comparison condition

Comparison condition dyads received “usual care,” defined as any dyadic or family communication, activities, or classes about sexual health that would occur in the context of regular family, school, and community life, irrespective of the study. They received baseline and follow-up surveys but no SSS exposure.

### Data collection

Data were collected using computer-assisted self-interviews on study laptop computers in the home, and follow-up data were collected at 93 (±5.9) days post-baseline.

### Primary outcome measures

The *frequency of parent–youth conversations* was assessed for eight sexual health topics in the past 3 months (Table 1).^39^ *Youth intentions* to engage in oral or vaginal sex, remain abstinent until the end of high school or until marriage, and use a condom (if sexually active) and *youth exposure to situations that could promote sexual activity* were assessed with items having had extensive pretesting with middle school aged adolescents.^20–22^ *Parents’ perceptions of their child’s intentions* regarding sexual activity were also assessed.

**Table 1.** Primary Outcome Measures

Construct	Youth			Parent		
	Variable^a^	Sample item	No. of items; Response options^b^ (Cronbach’s alpha^c^ as applicable)	Variable^a^	Sample item	No. of items; Response options^b^ (Cronbach’s alpha^c^ as applicable)
Parent–child communication about sex^d^	Frequency of communication about sex (developed)	In the past 3 months, how often have you and your caregiver talked about when to start having sex?	*N* = 8; 11-point (None to 10 or more times)	Frequency of communication about sex (developed)	In the past 3 months, how often have you and your child talked about when to start having sex?	*N* = 8; 11-point (None to 10 or more times)
Intentions regarding sex (youth)/Beliefs about child’s intentions regarding sex (parent)	Intention to have oral sex in the next year	How likely is it that you will have oral sex in the next year?	*N* = 1; 5-point (Not at all likely to definitely likely)	Beliefs about child’s intentions for oral sex	How likely is it that your child will have oral sex in the next year?	*N* = 1; 5-point (Not at all likely to definitely likely)
	Intention to have vaginal sex in the next year	How likely is it that you will have vaginal sex in the next year?		Beliefs about child’s intentions for vaginal sex	How likely is it that your child will have vaginal sex in the next year?	
	Intention to remain abstinent until the end of high school	How likely is it that you will remain sexually abstinent (that is, not have sex) from now until the end of high school?		Beliefs about child’s intentions to remain abstinent until high school	How likely is it that your child will remain sexually abstinent (that is, not have sex) from now until the end of high school?	
	Intention to remain abstinent until marriage	How likely is it that you will remain sexually abstinent (that is, not have sex) from now until marriage?		Beliefs about child’s intentions to remain abstinent until marriage	How likely is it that your child will remain sexually abstinent (that is, not have sex) from now until marriage?	
	Intention to use a condom	Do you intend to use a condom if you have sex in the next year?		Beliefs about child’s intentions to use condoms	How likely is it that your child will use a condom if they have sex in the next year?	
Risk behaviors^e^	Exposure to risky situations that could lead to sex^e^	In the past 3 months, how often have you invited a boyfriend or girlfriend to your home when an adult was not present?	*N* = 7; 7-point (Never to 6 or more times); (0.74)			

a Unless otherwise noted, measures come from the It’s Your Game…Keep It Real survey.^20–22^

b All items were coded so that a higher score indicated greater protection.

c Reliability indices were calculated using the analytical sample, limited to those who took the follow-up survey.

d Follow up survey only. Communication topics included: when to start having sex, birth control, condoms, AIDS or HIV, reproduction and having babies, physical and sexual development, STIs, and how to handle sexual pressure from friends or potential partners.

e Situations include: A party when alcohol is served, a friend’s house when adults are not present, having a boy/girlfriend home when an adult was not present, being in a car with someone who had been drinking, being alone with someone you are attracted to, being alone kissing and touching someone you really like, and laying down on a couch or bed alone with someone you really like.

HIV, human immunodeficiency virus; STI, sexually transmitted infection.

### Secondary outcome measures

*Psychosocial determinants of parent–child communication* comprised perceived quality of communication,^39^ communication ability,^39^ communication openness,^40^ self-efficacy for parent–child communication,^41^ and outcome expectations for communication (Table 2).^41^ General parent–child communication was assessed on dimensions of openness and problems.^42^

**Table 2.** Secondary Outcome Measures for Youth and Parents^1^

Construct	Variable^a^	Sample item	No. of items; response options^b^ (Cronbach’s alpha^c^ as applicable for youth / parent)
Communication about sex	Youth and parent		
Attitudes	Perceived quality of sex communication^37^	My caregiver doesn’t know enough about sexual topics like this to talk to me / I don’t know enough about sexual topics like these to talk to my child.	*N* = 10; 4-point *(Strongly disagree* to *strongly agree);* (0.76 / 0.69)
	Perceived ability of sex communication^37^	How would you rate your ability to communicate with your (caregiver/child) about sexual topics?	*N* = 1; 7-point *(Terrible* to *excellent)*
	Perceived openness about sex communication^38^	My caregiver tries to understand how I feel about sexual topics / My child feels comfortable asking me questions about sexual topics	*N* = 7; 4-point *(Strongly disagree* to *strongly agree);* (0.82) *N* = 9; 4-point *(Strongly disagree* to *strongly agree);* (0.85)
Self-efficacy	Self-efficacy to talk to parent/child about sex^39^	How sure are you that you can talk to your caregiver about how to use birth control? / How sure are you that you can explain to your child how to use birth control.	*N* = 16; 7-point *(Not sure at all* to *completely sure);* (Basic 0.95, Rel 0.91 / Basic 0.88, Rel 0.89)
Outcome expectations	Outcome expectations for talking with parent/child about sex^39^	If you talk with your caregiver about sexual topics you will feel responsible / If you talk with your child about sexual topics, you will feel like a responsible parent.	*N* = 15; 5-point *(Strongly disagree* to *strongly agree);* (Cog 0.80, emo 0.73, social 0.80) / (Cog 0.51, emo 0.81, soc 0.74)
Sexual behavior	Youth and parent		
Beliefs	Beliefs about sex	I believe that it is okay for people my age / my child’s age to have sex with a serious boyfriend or girlfriend	*N* = 4; 4-point *(Strongly disagree* to *strongly agree);* (0.85 / 0.78)
	Beliefs about abstinence	The best way for young people to avoid a sexually transmitted disease (STD) is to wait until they are married before they have sex.	*N* = 7; 4-point *(Strongly disagree* to *strongly agree);* (0.91 / 0.93)
Knowledge	Condom knowledge	Do condoms help a person keep from getting HIV, the virus that causes AIDS?	*N* = 6; 3-point *(No, Yes, Not sure);* (0.78 / 0.67)
	STI signs and symptoms knowledge	Mark whether you think the following are common signs of having an STI.	*N* = 7; 7 options (e.g., *Throwing up or vomiting, headache, no symptoms);* (NA^d^)
	HIV/STI knowledge	Some STIs put you at higher risk of getting infected with HIV.	*N* = 5; 3-point *(True, false, not sure);* (0.77/ 0.49)
General communication	Openness of communication^40^	I can discuss my beliefs with caregiver/ child without feeling restrained or embarrassed.	*N*-10; 5-point *(Strongly disagree* to *strongly agree*); (0.87)
	Problem communication^40^	There are topics I avoid discussing with my caregiver / child.	*N*-10; 5-point *(Strongly disagree* to *strongly agree);* (0.79)
	Youth only		
Perceptions of friends and parents	Perceived friends’ beliefs about sex	Most of my friends believe people should wait until they are older before they have sex	*N* = 4; 4-point *(Strongly disagree* to *strongly agree);* (0.83)
	Perceived parents’ beliefs about sex	My caregiver believes people my age should wait until they are older before they have sex	*N* = 4; 4-point *(Strongly disagree* to *strongly agree);* (0.68)
Self-efficacy	Sexual refusal self-efficacy	Could you stop this person you like if they wanted to have oral sex with you, if you did not want to?	*N* = 7; 4-point *(I definitely could not* to *I definitely could);*(0.93)
	Condom negotiation self-efficacy	Imagine that you have a boyfriend or girlfriend. Imagine that you and your boyfriend or girlfriend have been having sex but have not used condoms. You really want to start using condoms. How sure are you that you could tell your partner that you want to start using condoms?	*N* = 5; 3-point *(Not sure at all* to *Definitely sure);* (0.83)
Outcome expectations	Expectations about sex	If I had sex (e.g., vaginal or oral sex) at this time in my life, my caregiver would be embarrassed if I got pregnant or got a girl pregnant.	*N* = 13 neg n= 8 pos; 5-point *(Strongly disagree* to *strongly agree);* (0.89, neg), 0.93, pos)

a Unless otherwise noted, measures come from the It’s Your Game…Keep It Real survey.^20–22^

b All items were coded so that a higher score indicated greater protection.

c Reliability indices were calculated using the analytical sample, limited to those who took the follow-up survey.

d Knowledge scales were designed to examine specific information points targeted by the intervention, as opposed to comprehensive content domains.

*Psychosocial determinants of youth sexual initiation* comprised youth attitudes about sex and abstinence, knowledge regarding HIV/STIs and condoms, perceived friends’ and parents’ beliefs about sex, self-efficacy to refuse sex, self-efficacy to use condoms, and expectations regarding sex (Table 2).^20–22,39^ Parents were also assessed on their beliefs about sex and abstinence, and knowledge regarding HIV/STIs, and condoms.

### Usability

SSS was assessed on likeability, ease of use, duration, understandability, credibility, perceived impact, motivation to play, and appeal using youth and parent rating scales.^35^

### Demographic measures

Demographic measures included gender, age, grade, race/ethnicity, youth academic performance, parental education, household structure, household annual income, religiosity, and parenting style, adapted from other adolescent research.^20–22^ Items assessing youth sexual experience included ever having a boyfriend or girlfriend and ever engaging in oral and/or vaginal sex.^20–22^ Gaming experience included preferred platforms and frequency of play in the last 3 months.^43^

### Data analysis

Between-group differences in outcome variables from baseline to 3-month follow-up were evaluated using linear regression. Univariate regressions were run to assess between-group differences. Youth age at baseline, gender, ethnicity, and time between test administrations were entered into all models as covariates to adjust for any confounding variables. Missing values were left as missing and not imputed. All analyses were conducted using SAS 9.4.

## Results

### Sample characteristics

The analytic sample consisted of 83 parent–youth dyads. *Youth* were 12.9 (±1.1) years old, 54% male, 42.2% white, reported mainly A and B academic grades (75.9%), and most had never had a boyfriend or girlfriend (65%), vaginal (96.3) or oral sex (98.8%), nor any sexual health instruction in the last 3 months (65.9%) (Table 3). *Parents* were 44.4 (±5.8) years old, female (92.8%), of similar ethnicity to their youth, reported at least some college education (80.8%), were from two-parent households (73.5%), had over $50K income (72.3%), and were of Christian faith (86.1%). Youth reported that parents typically made decisions on their behalf (84.3%), often after asking their opinion (58.5%). Youth played games over 2 hours/week (69.6%), mostly on gaming consoles (35.8%) and phones (29.6%). Top-ranked genres were (in rank order): creative, fighting/shooter, adventure, casual, and simulation games. Parents played under 2 hours/week (87.4%), mostly on phones (56.3%). No significant demographic differences were observed across intervention and comparison groups. Top-ranked genres were (in rank order): casual, board, quiz, exercise, and educational games.

**Table 3.** Sample Characteristics of the Final Analytic Sample (*n* = 83 Parent–Youth Dyads)^a^

		Youth						Parents					
Construct	Variable	Total		Intervention		Comparison		Total		Intervention		Comparison	
		*n*	% / mean (±SD)	*n*	% / mean (±SD)	*n*	% / mean (±SD)	*n*	% / mean (±SD)	*n*	% / mean (±SD)	*n*	% / mean (±SD)
Age	Years	83	12.9 (1.1)	39	13 (1.1)	44	12.9 (1.2)	83	44.4 (5.8)	39	44.3 (6)	44	44.6 (5.6)
Gender	Male	45	54.2	19	48.7	26	59.1	6	7.2	2	5.1	4	10
	Female	38	45.8	20	51.3	18	40.9	77	92.8	37	94.9	40	90
Ethnicity	African American	24	28.9	10	25.6	14	31.8	24	28.9	10	26.3	14	32.6
	Hispanic	19	22.9	8	20.5	11	25.0	18	21.7	7	18.4	11	25.6
	Caucasian	35	42.2	19	48.7	16	36.4	39	47	21	55.3	18	41.9
	Other	5	6.0	2	5.1	3	6.8	0	0	0	0	0	0
Academic performance	Mostly A’s and B’s	63	75.9	31	79.5	32	72.7	60	72.3	27	69.2	33	75.0
	Mostly B’s and C’s	19	22.89	8	20.5	11	20.5	21	25.3	12	30.8	9	20.5
Dating behavior	Never had boyfriend/girlfriend	54	65.1	30	68.1	24	63.1	n/a					
Sexual experience	Never had vaginal sex	79	96.3	38	100	41	93.2						
	Never had oral sex	81	98.8	38	100	43	97.7						
Previous sexual health instruction	Ever	58	69.8	27	69.2	31	70.5						
	Last 3 mo.	28	34.1	13	33.3	15	34.8						
Parent education	At least some college	n/a						67	80.8	33	86.9	34	77.2
	Graduated high/tech school							13	15.6	5	11.1	8	18.2
Household structure	Both biological parents							55	66.3	24	61.5	31	70.5
	One biological parent							21	25.3	11	28.2	10	22.7
	Biological + step parent							6	7.2	3	7.7	3	6.8
Household income	<$50K							18	21.7	7	19.4	11	26.2
	≥$50K							60	72.3	29	80.6	31	74.1
Religiosity	Christian							74	86.1	33	80.5	41	91.1
Parenting style	Parent decides	22	26.8	11	28.2	11	25.6	n/a					
	Parent asks opinion /decides	48	58.5	22	56.4	26	60.5						
	Child asks opinion/decides	9	10.9	4	10.3	5	11.6						
	Child decides	3	3.7	2	5.1	1	2.3						
Gaming genre (top 3 ranked)^b^	1	83	Creative	39	Creative	44	Fight/ shoot	83	Casual	39	Casual	44	Casual
	2		Fight/shoot		Adventure		Creative		Board		Board		Board
	3		Adventure		Fight/ shoot		Casual		Quiz		Quiz		Quiz
Gaming platform (top 3)	Phone	24	29.6	10	26.3	14	32.6	40	56.3	21	67.7	19	47.5
	Gaming console	29	35.8	11	28.9	18	41.7	6	8.5	2	6.5	4	10.0
	Tablet/pad	11	13.6	6	15.8	5	11.6	11	15.5	3	9.7	8	20.0
Gaming frequency (past 3 mo.)	>2 h/week	48	71.6	30	78.9	28	65.1	26	36.6	9	29.0	13	42.5
	<2 h/week	21	25.9	7	18.4	14	32.6	33	46.5	16	51.6	17	42.5
	None	2	2.5	1	2.6	1	2.3	12	16.9	6	19.4	6	15.0

a No significant demographic differences were observed across intervention and comparison groups.

b Game categories comprised: Adventure, board, casual, creative, educational, exercise, fighting/shooter, multiplayer, artificial/virtual pets, quiz, role-playing, simulation, sports, strategy.

### Primary outcomes

#### Parent–child frequency of communication about sex

Youth exposed to SSS reported increased discussion regarding when sex should be initiated (*P* = 0.0012) (Table 4). Their parents reported increased discussion regarding birth control (*P* = 0.0014), condoms (*P* = 0.034), and handling peer pressure (*P* = 0.0028). *Youth intentions and risk exposure* did not change significantly. Parents reported an increase in their youth’s intentions to use a condom if they engaged in sex (*P* < 0.05), but that change in other youth’s intentions was not significant.

**Table 4.** Intervention Effects on Primary and Secondary Outcomes by 3-Month Follow-up among the Analytic Sample (*n* = 83)^a^

	Youth					Parent				
Outcome measure^b^	*n^c^*	Beta^d^	SE	95% CI		*n* ^*c*^	Beta^d^	SE	95% CI	
Communication, intentions, and risk avoidance										
Parent–youth communication about sex										
Comm re. sex: when to start having	78	**1.92**	0.57*	**0.81**	**3.02**	83	0.42	0.52	−0.59	1.44
Comm re. sex: birth control	79	0.21	0.28	−0.34	0.75	83	**1.1**	0.33^**^	**0.45**	**1.75**
Comm re. sex: condoms	79	0.07	0.41	−0.72	0.87	83	**0.74**	0.34^***^	**0.07**	**1.41**
Comm re. sex: AIDS/HIV	79	0.31	0.44	−0.55	1.18	83	0.49	0.42	−0.33	1.31
Comm re. sex: Reproduction	79	0.8	0.52	−0.22	1.82	83	0.19	0.56	−0.91	1.29
Comm re. sex: Development	79	−0.26	0.49	−1.21	0.68	83	−0.23	0.63	−1.46	1.005
Comm re. sex: STDs	79	0.54	0.46	−0.35	1.44	83	0.83	0.46	−0.07	1.73
Comm re. sex: Handling pressure	79	0.67	0.63	−0.56	1.89	83	**1.14**	0.37^**^	**0.41**	**1.87**
Parent–youth general communication:										
Openness of communication	79	0.35	1.21	−2.06	2.75	83	0.94	0.7	−0.46	2.34
Problem communication	76	−0.96	1.29	−3.54	1.61	83	**−2.72**	0.91^*^	**−4.55**	**−0.90**
Youth intentions toward sex^e^										
Intention to use a condom	73	−0.17	0.29	−0.73	0.34	72	**0.59**	0.29^***^	**0.02**	**1.16**
Exposure to risky situations	78	−0.11	0.06	−0.23	0.01	n/a				
Determinants of parent–youth communication about sex										
Attitudes and beliefs										
Perceived quality of sex communication with parent or child	69	1.07	0.81	0.55	2.68	79	0.63	0.61	−0.59	1.85
Perceived ability of sex communication with parent or child	75	0.07	0.07	−0.08	0.21	83	**0.44**	0.20^***^	**0.04**	**0.85**
Perceived openness about sex communication with parent or child	66	**0.26**	0.12^**^	**0.02**	**0.48**	82	0.18	0.07	0.03	0.32
Self-efficacy										
Self-efficacy to talk to parent [or child] about sex (Total scale)	67	**0.78**	0.37^*^	**0.02**	**1.53**	80	0.07	0.12	−0.17	0.31
Self-efficacy to discuss relationships	69	0.12	0.39	−0.68	0.91	82	−0.13	0.1	−0.33	0.08
Self-efficacy to discuss practical information	73	0.01	0.19	−0.38	0.4	83	0.24	0.12	−0.00	0.47
Outcome expectations (OE)										
OE for talking with parent or child about sex (cognitive)	75	0.17	0.19	−0.23	0.57	82	0.05	0.09	−0.14	0.24
OE for talking with parent or child about sex (emotional)	75	−0.25	0.14	−0.53	0.04	83	−0.18	0.12	−0.42	0.05
Determinants of youth sexual behavior^f^										
Attitudes and beliefs										
Beliefs about sex	78	0.15	0.11	−0.06	0.37	83	0.02	0.05	−0.09	0.13
Beliefs about abstinence	75	0.14	0.12	−0.10	0.38	80	0.22	0.12	−0.01	0.45
Knowledge										
Condom knowledge	77	**0.19**	0.07^*^	**0.06**	**0.32**	83	−0.00	0.03	−0.05	0.05
HIV/STI knowledge	77	**0.28**	0.06^*^	**0.16**	**0.39**	83	−0.04	0.02	−0.08	0.01
STI signs and symptoms knowledge	63	0.06	0.05	−0.04	0.15	83	−0.03	0.03	−0.09	0.02
Perceptions of friends and parents										
Perceived friends’ beliefs about sex	76	−0.11	0.12	−0.34	0.12	n/a				
Perceived parents’ beliefs about sex	76	**0.198**	0.08^*^	**0.04**	**0.35**					

The bold data represents significant results.

a Models are adjusted for youth age, youth gender, youth ethnicity, and time between measures.

b All psychosocial variables are coded as protective factors.

c Sample sizes vary due to missing data.

d Beta = Effect size difference in adjusted mean.

e Between-group difference for youth intentions to engage in oral or vaginal sex, remain abstinent until the end of high school or until marriage, and to use a condom (if sexually active) were not statistically significant.

f Self-efficacy and outcome expectations were NS.

* *P* < 0.05.

** *P* < 0.01.

*** *P* < 0.001.

CI, confidence interval; SE, standard error.

### Secondary outcomes

#### Psychosocial determinants of parent–child communication

Youth exposed to SSS reported significantly more openness about sexual health communication with their parent (*P* = 0.031) and self-efficacy to talk to their parents about sex (*P* = 0.043) (Table 4). Parents reported greater ability to communicate with their youth about sex (*P* = 0.017) and significantly reduced problem communication (*P* = 0.003). *Psychosocial determinants of youth sexual behavior:* Youth exposed to SSS reported significantly greater knowledge about condoms (*P* = 0.005) and HIV/STIs (*P* ≤ 0.001) as well as more positive perceptions of their parents’ beliefs about sex (*P* = 0.013). Other determinants were not significant.

### Intervention exposure

By 3-month follow-up, 24 dyads (60%) completed SSS, four dyads (10%) finished over 50% but did not complete SSS, and 12 dyads (30%) finished less than 50% of SSS. Delays in game play were attributed to competing school obligations and standardized testing, competing family activities, and program “bugs” (e.g., connectivity drops and program freezes).

### Usability

Most parents in the intervention condition (70%) rated SSS favorably on all usability parameters except game duration. Most youth (70%) agreed that SSS was easy to use and would be impactful. Fewer youth (<70%) agreed that SSS was of appropriate duration, motivationally appealing, likable, and accurate. More youth liked the dojo activities (72%) and battles (67%), while fewer liked the PEP talks (50%). Most parents (66%) and youth (78%) required no help to play SSS.

## Discussion

This study is among the first to report on an IGG for sexual health education.

Results suggest utility in facilitating parent–youth sexual health communication and enhancing psychosocial determinants (self-efficacy toward parental communication, knowledge, and perceived norms) that is commensurate with findings from previous computer-based and gaming interventions to promote sexual health that report small to moderate effects on sexual behavior, sexual health knowledge, and safer sex self-efficacy and intentions.^13,28,36^

Increased communication about sexual initiation and risk reduction (birth control, condoms, and handling pressure) and youths’ improved perceptions of communication openness and increased confidence to talk to their parent about sexual health topics is encouraging. Youth traditionally report discomfort in discussing sexual health or disclosing sensitive issues with their parents.^25,44–46^ IGG features, including a common educational experience, shared expectations for communication, and a game dynamic contingent on completion of PEP talks, may have contributed to these results.

Increasing youth confidence to discuss sexual health with their parents does little if parents themselves lack a similar confidence or are unprepared or untrained. The intergenerational dyadic approach offers advantages in this regard. Parents exposed to SSS reported greater ability to engage in communication and reported less problem communication, suggesting some mitigation of parents’ traditional feelings of discomfort and avoidance of these discussions.^25,45–49^ Parents’ increased perceptions that their youth would use a condom if they engaged in sex and youth’s enhanced knowledge about condoms and HIV/STIs were consistent with the increased parent–youth communication on this topic. Increased communication frequency did not prevent PEP talks from receiving the lowest likability rating from youth. These discussions were still not regarded as particularly “fun.”

The impact on psychosocial determinants of condom uses and increased positive perceptions of parents’ beliefs about sex were consistent with previous studies.^11,20–22^ Though not statistically significant, changes in the remaining determinants were in the predicted direction of protection for dyadic communication, delayed sexual initiation, and risk reduction. This suggests the potential for SSS to demonstrate greater effectiveness with increased analytic power.

Numerous national and professional guidelines advocate the implementation of sexual health education in schools and clinics.^50–54^ However, implementation of recommendations and sexual health education content and duration is inconsistent and often inadequate.^55^ Parents remain a vital and consistent resource to facilitate life-skills training of their youth.^56^ Policy- and decision-makers can support the inclusion of parents in this critical role by funding research into the development and dissemination of IGG best practice models in school, clinic, community, and home settings, and supporting evaluation of long-term outcomes of this strategy.

Results need to be interpreted in the context of study limitations. The randomized design, used to mitigate threats to internal validity, was not blinded by condition, so it was subject to possible compensatory bias. The short-term follow-up limited the ability to adequately assess behavioral changes such as risk avoidance or delayed sexual initiation. Using recall to assess communication frequency can introduce inconsistency because parents generally report higher frequencies,^18,57^ and report being more direct, less avoidant, and less controlling in conversations about sexuality than youth.^44,58^ SSS accommodates a passive, supportive gatekeeper role for parental players that represents one exemplar of an IGG, so findings are subject to mono-operational bias and may not generalize to all IGGs.^59^ This study did not assess verbal and non-verbal communication mechanics and quality, including content, disclosure, depth, and flow. The degree to which discussions were superficial (e.g., simply handing over the unlock code) or “deep” (e.g., exchanging personal perspectives and experiences) remains open to further research.^60–62^ Despite positive usability ratings, many youths (30%) did not complete half of SSS, suggesting that it may be insufficiently motivating for all youths. This is important as exposure to core sexual health content needs to be sufficient to expect behavioral outcomes.^35^ To more fully assess the fidelity of the parent website, tracking metrics are recommended. The study required greater statistical power to enable assessment of covariates (e.g., intervention intensity, duration, tailoring, and context), or moderating factors (e.g., preferred game genres or learning styles) that may have contributed to the findings and present a potential focus for future IGG research.

## Conclusion

This study is among the first to rigorously evaluate an in-home IGG for sexual health education and demonstrated the utility of this strategy to positively affect parent–youth communication by short-term follow-up. The findings of this study are specific to the SSS but contribute to evidence for intergenerational gaming to increase parental involvement and enhance the reach and effectiveness of sexual health education. Further investigation of longer-term behavioral impact is indicated.

## Authors’ Contributions

R.S.: Conceptualization, methodology, validation, investigation, resources, writing (original draft and review and editing), visualization, supervision, and funding acquisition. C.M.: Methodology, validation, and writing (review and editing). M.P.: Methodology, validation, and writing (review and editing). R.C.A.: Formal analysis and data curation. S.D.: Supervision and project administration. D.S.M.: Validation. S.T.E.: Methodology, validation and writing (review and editing). J.M.W.: Validation. E.B.: Formal analysis and data curation; L.A.: Investigation. P.C.: Investigation. H.-Y.S.: Investigation. A.S.: Investigation. J.M.: Conceptualization, methodology, resources, supervision, and funding acquisition.

## Data Sharing Statement

The authors intend to make the study data available to other researchers subsequent to analysis by the research team. Analytic methods and study materials are available upon request to the corresponding author.
